# Association of serum leptin with serum C-reactive protein in hemodialysis patients

**Published:** 2012-07-01

**Authors:** Ebrahim Rastegari, Hamid Nasri

**Affiliations:** ^1^Section of Hemodialysis, Shahrekord University of Medical Sciences, Shahrekord, Iran

**Keywords:** Hemodialysis, End-stage renal failure, Leptin, C-reactive protein

## Abstract

**Introduction:** Recent investigations have shown that leptin is cleared principally by the kidney.

**Objectives:** To examine whether and how in patients on hemodialysis the level of C-reactive protein level correlate with serum leptin.

**Patients and Methods:** The total patients were 36. The mean patients’ age were 46 (16) years. The median length of the time patients were on hemodialysis were 19 months.

**Results:** The mean serum C-reactive protein was 8.7 (6.6) mg/l (median: 8 mg/l). The mean serum leptin was 9.4 (14) ng/ml (median: 5.75 ng/ml). In this study we found a significant inverse correlation of serum leptin with serum C-reactive protein (r= -0.57, p= 0.041) was seen.

**Conclusion:** Our data supports, the positive effect of leptin on nutrition and support the theory of protective effects (reverse epidemiology) of leptin in hemodialysis patients.

**Implication for health policy/practice/research/medical education:**

In a study on 36 stable hemodialysis patients, we found, the inverse correction of leptin with C-reactive protein level. Our data supports, the positive effect of leptin on nutrition and support the theory of protective effects (reverse epidemiology) of leptin in hemodialysis patients.

## Introduction

Recent investigations have shown that leptin is cleared principally by the kidney. Serum leptin levels are increased in patients with chronic kidney failure and those undergoing hemodialysis (1,2) and it has been thought that high leptin value may contribute to uremic anorexia and malnutrition (4,5). In the general population leptin is considered an “appetite inhibitor”, however in contrast to preliminary findings, its role in chronic renal disease and hemodialysis patients is not completely found. While serum leptin is generally elevated in chronic kidney failure and hemodialysis patients, however, some other studies have not been shown to be a cause of uremia-related anorexia (6,7). More recent investigations in hemodialysis patients suggest a paradoxically inverse correction between higher serum leptin and improved markers of nutritional condition (6,7), a finding that is indicative of reverse epidemiology in this population (8). In fact, leptin, similar to serum albumin, has been reported to be a negative acute phase reactant in end-stage kidney failure (7). C-reactive protein (CRP) is an acute phase protein whose synthesis in the liver. CRP is regulated by different cytokines, especially interleukin 6 (IL-6). Plasma level of CRP in the absence of active disease are low, but can rise up to 1000-fold in patients with an inflammatory reaction. In addition to being a marker of inflammation, CRP itself may have pro-inflammatory properties while it can activate the complement system (8,9). Therefore elevated plasma concentrations of CRP, a sensitive marker of underlying systemic inflammation (10-12). Serum CRP value have also been shown to be significantly elevated in patients on hemodialysis (13,14) and suggests chronic inflammation. Serum CRP, is a sensitive and independent marker of malnutrition (15). According to the present data, investigations concerning the association of CRP with serum leptin needs further examination.

## Objectives

The aim this study was to elucidate whether and how in patients on hemodialysis the serum value of CRP as the marker of inflammation correlate with serum leptin.

## Patients and Methods

### Patients

This cross-sectional study was conducted on patients with end-stage kidney disease, who were undergoing regular hemodialysis. The study was conducted in hemodialysis section of Shahrekord University of Medical Sciences.

### Laboratory assessments

Blood samples were obtained after an overnight fast. For patients, Levels of serum CRP, leptin (normal range of values for males is 3.84 (1.79) and for females is 7.36 (3.73) ng/ml were measured by enzyme-linked immunosorbent assay (ELISA). Duration and doses of hemodialysis treatment were calculated from the patients’ records. The duration of each hemodialysis session was 4 hours. For the efficacy of hemodialysis the urea reduction rate (URR) was calculated from pre- and post-blood urea nitrogen (BUN) data (16). Body mass index (BMI) calculated using the standard formula (postdialyzed weight in kilograms/height in square meters; kg/m^2^) (17).

### Ethical approval

All patients signed the consent form for participation in this study. Research study was approved by the ethics committee of Shahrekord University of Medical Sciences, Iran.

### Statistical analysis

Results are expressed as the mean (SD) and median values. Statistical correlations were assessed using partial correlation test. For leptin correlation, the logarithm of serum leptin values was used. All statistical analyses were performed using SPSS (version 12.00). Statistical significance was determined at a p-value<0.05.

## Results

Study patients were 36. Table 1 shows patients’ data. The mean patients’ age were 46 (16) years. The mean length of the time patients had received hemodialysis was 32 (36) months. The mean serum CRP was 8.7 (6.6) mg/l. The mean serum leptin was 9.4 (14) ng/ml (median: 5.75 ng/ml). In this study a significant inverse correlation of serum leptin with serum CRP (r= -0.57, p= 0.041) was seen.

**Table 1:** Data of patients.

Total patients= 36	Minimum	Maximum	Mean (SD)	Median
Age (years)	16	80	46(16)	43
DH* ( months)	2	156	32(36)	19
CRP mg/l	3	40	8.7(6.6)	8
Mg mg/dl	1.6	3.5	2.5±0.4	2.4

## Discussion

In this study we detected a significant inverse correlation of serum leptin with CRP value. The increased levels of leptin in hemodialysis patients are mainly due to retention of the hormone. Recent studies in maintenance dialysis patients suggest a paradoxically inverse association between higher serum leptin and improved markers nutritional status (6,7), a finding that is in accord with the theory of reverse epidemiology (8,18-21). Indeed, leptin, similar to serum albumin, has been reported to be a negative acute phase reactant in hemodialysis patients (7). While, in the general population, leptin is considered an “appetite inhibitors”, its role in hemodialysis patients is somewhat different. Serum leptin is generally elevated in hemodialysis, but this has not been shown to be related to anorexia. In contrast leptin has been shown to act synergistically with erythropoietin to stimulate the end-stage colony-forming-unit erythroid in humans (22).

## Conclusion

Our data indirectly supports the some previous investigations regarding the hypothesis that in patients on hemodialysis, leptin is a negative acute phase reactant and leptin might have a positive effects on nutrition and support the theory of protective effects of leptin in hemodialysis patients.

## Author’s contributions

ER and HN wrote the manuscript equally.

## Conflict of interests

The authors declared no competing interests.

## Ethical considerations

Ethical issues (including plagiarism, misconduct, data fabrication, falsification, double publication or submission, redundancy) have been completely observed by the authors.

## Funding/Support

This study was supported by a grant from Shahrekord University of Medical Sciences.
